# Organization of left-right coordination of neuronal activity in the mammalian spinal cord locomotor CPG: insights from computational modeling

**DOI:** 10.1186/1471-2202-15-S1-O10

**Published:** 2014-07-21

**Authors:** Ilya A Rybak, Natalia A Shevtsova, Adolfo E Talpalar, Sergey N Markin, Ronald M Harris-Warrick, Ole Kiehn

**Affiliations:** 1 Department of Neurobiology and Anatomy, Drexel University College of Medicine, Philadelphia, PA 19129, USA; 2 Department of Neuroscience, Karolinska Institute, Stockholm, 17177, Sweden; 3 Department of Neurobiology and Behavior, Cornell University, Ithaca, NY 14853, USA

## 

Different gaits of locomotion in mammals are based on the appropriate coordination of neuronal activity in the spinal cord controlling movements of left and right limbs. This left-right coordination is provided in the spinal cord by the commissural interneurons (CINs) whose axons cross the midline and affect neural circuits on the contralateral side of the cord. Several types of CINs have been genetically identified, including the excitatory V3 CINs and the inhibitory (V0_D_) and excitatory (V0_V_) V0 CINs. Talpalar et al. [1] recently demonstrated that (a) ablation of both V0 CIN types leads to a left-right synchronized, “hopping” activity at all locomotor frequencies, whereas (b) selective ablation of the excitatory V0_V_ CINs maintains alternation at low frequencies but switches to synchronized activity at high frequencies while (c) ablation of only the inhibitory V0_D_ CINs leads to a lack of left–right alternation at low frequencies, but maintains alternation at high frequencies. The genetically identified, ipsilaterally projecting excitatory V2a interneurons are recruited with an increase in locomotor speed [2] and contribute to left-right alternation at high locomotor frequencies [3,4]. Our objective was to construct and analyze a computational model of the bilaterally interacting central pattern generators (CPGs) that could reproduce and explain these findings.

In our model, the CPG on each side of the cord consisted of flexor and extensor half-centers. Each neural population, including the half-centers and CIN populations, consisted of 50-200 neurons modeled in the Hodgkin-Huxley style. The intrinsic bursting of CPG neurons was based on a persistent sodium current in these neurons.

During model construction, we assumed that left-right coordination of activity depends on the balance between the three CIN pathways providing interactions between the left and right CPGs: the V3-mediated pathway that supports left-right synchronization and the V0_D_- and V0_V_- mediated pathways that provide left-right alternation. The activity of each (left and right) inhibitory V0_D_ population was driven by the ipsilateral flexor half-center. The recruitment of V0_D_ neurons was progressively reduced with an increase in locomotor speed, because of the reduction of burst amplitude. The left and right V0_V_ pathways could be organized in two ways: (1) the V0_V_ activity on each side was driven by the ipsilateral flexor half-center and its action on contralateral circuits was mediated by an inhibitory population, or (2) the V0_V_ activity was driven by the ipsilateral extensor half-center and it excited the contralateral circuits. In any case, the V0_V_ activation was mediated by the ipsilateral V2a neurons progressively recruited with increasing locomotor speed.

The model demonstrates: (1) a left-right alternating pattern under control conditions; (2) a synchronized hopping pattern at any frequency after removing both the V0 populations; (3) a synchronized pattern at low frequencies with alternation at high frequencies after removing the V0_D_ populations; (4) an alternating pattern at low frequencies with synchronized hopping at high frequencies after removing either V0_V_ or V2a populations. The model closely reproduces and suggests an explanation for the experimental data of Talpalar et al. [1], Zhong et al. [2], and Crone et al. [3,4], proposes the organization of commissural interactions in the spinal cord defining the left-right alternation at different locomotor speeds, and generates predictions for future experimental investigations.

## References

[B1] Talpalar AE Bouvier J Borgius L Fortin G Pierani A Kiehn O Dual-mode operation of neuronal networks involved in left-right alternation Nature 2013 15 7460 85 88 10.1038/nature12286 23812590

[B2] Zhong G Sharma K Harris-Warrick RM Frequency-dependent recruitment of V2a interneurons during fictive locomotion in the mouse spinal cord Nat Commun 2011 15 274 10.1038/ncomms1276 21505430 PMC3597081

[B3] Crone SA Quinlan KA Zagoraiou L Droho S Restrepo CE Lundfald L Endo T Setlak J Jessell TM Kiehn O Sharma K Genetic ablation of V2a ipsilateral interneurons disrupts left-right locomotor coordination in mammalian spinal cord Neuron 2008 15 1 70 83 10.1016/j.neuron.2008.08.009 18940589

[B4] Crone SA Zhong G Harris-Warrick R Sharma K In mice lacking V2a interneurons, gait depends on speed of locomotion J Neurosci 2009 15 21 7098 109 10.1523/JNEUROSCI.1206-09.2009 19474336 PMC2731420

